# Making Co-Enrolment Feasible for Randomised Controlled Trials in Paediatric Intensive Care

**DOI:** 10.1371/journal.pone.0041791

**Published:** 2012-08-03

**Authors:** Katie Harron, Twin Lee, Tracy Ball, Quen Mok, Carrol Gamble, Duncan Macrae, Ruth Gilbert, on behalf of CATCH. team

**Affiliations:** 1 MRC Centre for Epidemiology of Child Health, Institute of Child Health, University College London, London, United Kingdom; 2 Paediatric Intensive Care Unit, Great Ormond Street Hospital, London, United Kingdom; 3 Clinical Trials Research Centre, University of Liverpool, Liverpool, United Kingdom; 4 Paediatric Intensive Care Unit, Royal Brompton Hospital, London, United Kingdom; Nottingham University, United Kingdom

## Abstract

**Aims:**

Enrolling children into several trials could increase recruitment and lead to quicker delivery of optimal care in paediatric intensive care units (PICU). We evaluated decisions taken by clinicians and parents in PICU on co-enrolment for two large pragmatic trials: the CATCH trial (CATheters in CHildren) comparing impregnated with standard central venous catheters (CVCs) for reducing bloodstream infection in PICU and the CHIP trial comparing tight versus standard control of hyperglycaemia.

**Methods:**

We recorded the period of trial overlap for all PICUs taking part in both CATCH and CHiP and reasons why clinicians decided to co-enrol children or not into both studies. We examined parental decisions on co-enrolment by measuring recruitment rates and reasons for declining consent.

**Results:**

Five PICUs recruited for CATCH and CHiP during the same period (an additional four opened CATCH after having closed CHiP). Of these five, three declined co-enrolment (one of which delayed recruiting elective patients for CATCH whilst CHiP was running), due to concerns about jeopardising CHiP recruitment, asking too much of parents, overwhelming amounts of information to explain to parents for two trials and a policy against co-enrolment.

Two units co-enrolled in order to maximise recruitment to both trials. At the first unit, 35 parents were approached for both trials. 17/35 consented to both; 13/35 consented to one trial only; 5/35 declined both. Consent rates during co-enrolment were 29/35 (82%) and 18/35 (51%) for CATCH and CHiP respectively compared with 78% and 51% respectively for those approached for a single trial within this PICU. The second unit did not record data on approaches or refusals, but successfully co-enrolled one child.

**Conclusions:**

Co-enrolment did not appear to jeopardise recruitment or overwhelm parents. Strategies for seeking consent for multiple trials need to be developed and should include how to combine information for parents and patients.

## Introduction

Rapid changes in practice result in a constant stream of drugs, procedures and devices that need to be evaluated in critically ill children. Many treatments and interventions used in paediatric intensive care (PICU) have no evidence base or are extrapolated from results of adult studies. For example, up to 90% of drugs prescribed to children in hospital are unlicensed and have either not been evaluated at all in children or for the specific condition for which the drug is used, and 67% of children receive drugs prescribed in an unlicensed or ‘off-label’ manner [1], [2], [3], [4]. Huge assumptions are made when drugs established in adult practice are used in children without supportive paediatric trials [5].

The growing expectation of evidence-based practice has led to improved infrastructure to support clinical trials in children in the UK. For example, national audit data collated by the Paediatric Intensive Care Audit Network (PICANet) can help to define eligible patient populations when planning multi-centre trials and assist with data capture [6]. More recently, the NIHR Medicines for Children Research Network (MCRN) was established to help increase the evidence base by improving the co-ordination, speed and quality of randomised controlled trials of medicines for children and young people by facilitating the acknowledged bureaucratic burden involved in establishing trials in the UK and encouraging pharmaceutical investment [7].

As the demand for trials in PICU continues to increase, concurrent studies need to make room for each other and minimise the burden of involvement on patients, parents and staff. At the time of writing, there were 145 studies listed on the UK Clinical Research Network Study Portfolio (http://public.ukcrn.org.uk) as actively recruiting children. Large sample sizes are required to adequately assess new treatments and interventions (the two trials included in this study aimed to recruit 1500 and 1200 children each) yet PICU has a finite capacity, with a total of 18,905 admissions nationally in 2010 [8]. Additionally, recruitment in PICU is particularly difficult as parents and children are already placed in a stressful situation [9].

Enrolling children into several trials at the same time can potentially make recruitment more efficient [10]. Co-enrolment has been successful in clinical trials for critically ill children in Africa and is feasible and acceptable for studies of AIDS treatments in children [11], [12], [13], [14]. A tri-nation survey found that 52% of clinicians or research coordinators had enrolled an ICU patient into more than one ICU study in the past year, and that respondents working in paediatric compared with adult critical care were more likely to endorse co-enrolment [15]. However, 13% stated that they would not offer enrolment of a patient into two randomized trials under any circumstances [15]. The stress researchers feel when recruiting in PICU may be compounded with more than one trial [16].

Ethics requirements are perceived as a barrier to co-enrolment. However, there are no explicit ethics committee guidelines that prohibit or discourage recruitment of children into more than one trial [17]. The MRC states that participation in more than one research study might be potentially beneficial both for the children involved and for the research, as long as the potential benefits and risks of doing so are carefully explored and clearly understood by the child and family [18].

No reports have yet been published of the practical issues arising when PICU clinicians are asked to co-enrol patients into two randomised controlled trials. We report PICU and parental decisions about participating in two PICU trials and determine the impact of co-enrolment on recruitment rates.

## Methods

We evaluated co-enrolment for two concurrent trials targeted at similar populations in PICU:

1. CATCH (CATheters in CHildren HTA project ref 08/13/47 ISRCTN34884569)
2. CHIP (Control of Hyperglycaemia in Paediatric intensive care: ISRCTN 61735247, http://www.controlled-trials.com)

Although other trials (e.g. SLEEPs, OXIC-2 and THERMIC-2) were recruiting in PICU at the same time, our study focuses only on CATCH and CHiP, as these are the largest PICU trials to date and have a large recruitment overlap.

Ethics statement: Written informed consent was obtained for all participants in CATCH and CHiP. Co-enrolment was permitted in both trial protocols. CATCH was approved by the South West Medical Research Ethics Committee (MREC) on 19/02/2010 (ref: 09/H0206/69). CHiP was approved by Brighton East REC on 01/06/2007 (ref: 07/Q1907/24).

### PICU Decisions

We recorded the period of trial overlap for all PICUs taking part in both CATCH and CHiP. We examined reasons for PICU clinicians deciding whether or not to co-enrol children into both studies.

### Parental Decisions

We examined parental decisions on co-enrolment to CATCH and CHiP by measuring whether being approached for co-enrolment affected recruitment rates, and collecting any reasons for refusal to consent. We compared recruitment rates for parents given the opportunity to co-enrol with consent rates for parents approached for one trial only. We retrieved data on the number of children who were eligible, approached and consented and collected reasons for refusal by parents of children approached for both trials. The CATCH protocol states that research nurses should routinely ask for reasons for non-consent but make clear to the parents/legal guardian that they do not have to provide a reason unless happy to do so. We also recorded research nurses’ experiences in seeking consent for two concurrent trials.

### Consent Process

CATCH is a multicentre 3-arm randomised controlled trial that aims to determine the effectiveness of heparin coated or antibiotic impregnated central venous catheters (CVCs) compared with standard CVCs for preventing hospital acquired blood stream infection in children admitted to PICU. Children were randomised to either standard, heparin-coated or antibiotic-impregnated CVCs, all of which are in routine use in PICU. The trial aims to recruit 1200 children under 16 years of age admitted to one of the 12 trial PICUs between November 2010 and September 2012.

CHiP is the largest PICU trial completed to date. It aimed to determine whether strict control of blood glucose using insulin in children admitted to PICU reduced mortality, morbidity and/or the use of healthcare resources compared with standard practices. Children were randomised to receive either standard care or tight glycaemic control. 1384 children under 16 years of age were recruited between May 2009 and August 2011.

Co-enrolment into the two trials was discussed with the research network and allowed in both protocols (available for CATCH at www.hta.ac.uk/1867; for CHiP at www.chip-trial.org.uk). Strategies for seeking consent to both trials were agreed with the chief investigators of both trials. Due to pressure to meet the recruitment target for CHiP it was agreed that patients eligible for both CATCH and CHiP would be approached for CHiP before being given any information about CATCH. In this way, units could open for CATCH without any potential detriment to CHiP recruitment.

**Figure 1:**
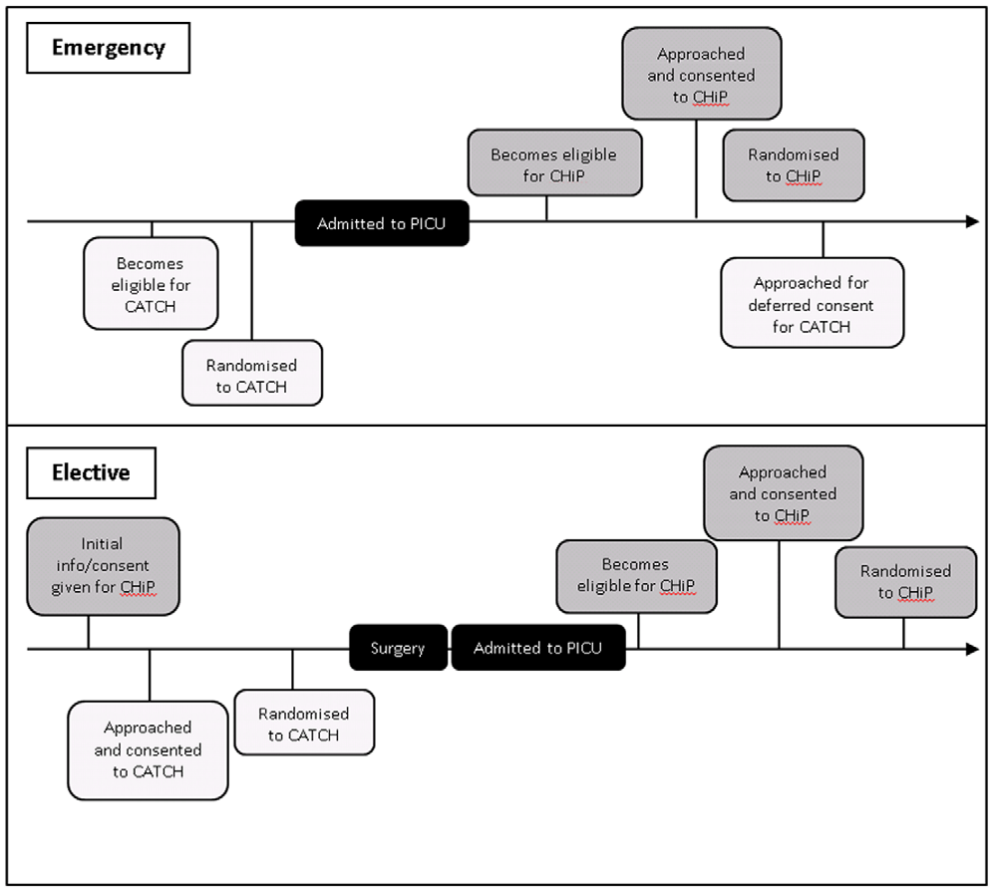
Timelines for eligibility, consent and randomisation for elective and emergency admissions for CATCH and CHiP.

Both trials recruited from elective and emergency admissions. Timelines for eligibility, consent and randomisation for elective and emergency patients are shown in Figure 1. For emergency admissions eligible for CHiP, consent was sought prior to the intervention. For emergency admissions in CATCH, children were firstly randomised to a trial CVC to avoid any delay in treatment and deferred consent was obtained once the patient was stabilised, usually within 48 hours of CVC insertion. An amendment to the Medicines for Human Use (Clinical Trials) Regulations in 2006 (UK SI 2006 No 2984) allows patients to be included in a trial before consent has been obtained if urgent treatment is required, and CATCH is the first UK trial to adopt deferred consent for children.

For elective surgical patients, consent was obtained prospectively for both trials. For CATCH, children were randomised before surgery. For CHiP, elective children did not become eligible until admitted to PICU after surgery. In these cases, parents would be approached for CATCH before becoming eligible for CHiP.

## Results

### PICU Decisions

**Figure 2:**
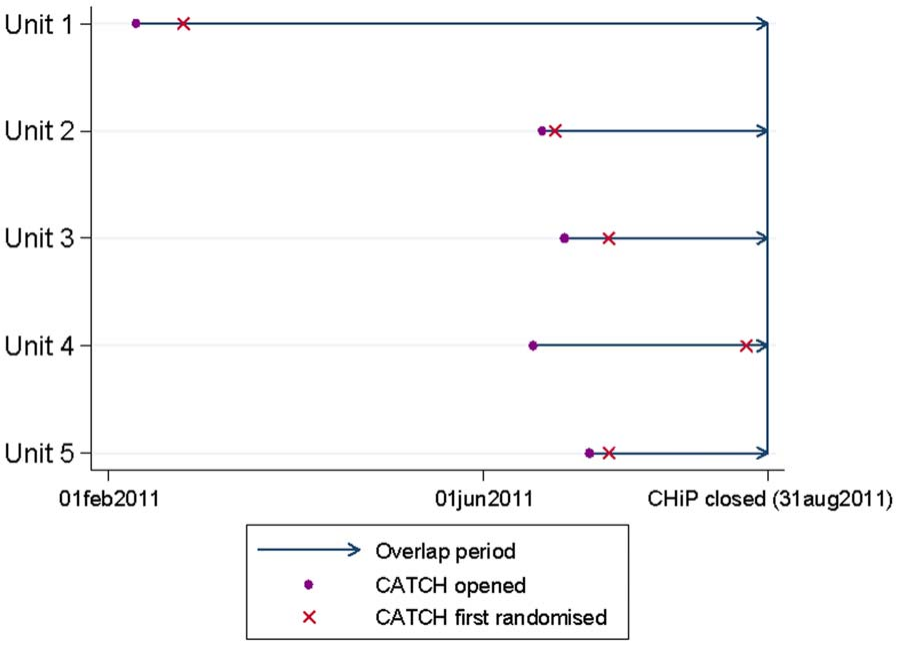
Period of trial overlap for PICUs recruiting to CATCH and CHiP during the same time period.

Five PICUs had the opportunity to recruit to both CATCH and CHiP. Only two units decided to adopt co-enrolment for CATCH and CHiP (Units 1 and 2 in Figure 2). These two units chose to co-enrol children to both trials so that recruitment for both trials could be maximised. Three units (Units 3 to 5 in Figure 2) decided not to allow children to be recruited into both trials. Of these three, one unit delayed recruiting elective patients for CATCH until CHiP had closed, resulting in a loss of six recruiting weeks for this unit. Reasons given for deciding not to allow co-enrolment were concerns about jeopardising recruitment targets for CHIP (the trial was scheduled to close in August 2011), asking too much of parents due to the overwhelming amounts of information to explain to parents for two trials and the already stressful situation of having a child in need of intensive care. One PICU had a policy against co-enrolment.

### Parental Decisions

Of the two units that adopted co-enrolment, Unit 1 recorded the number of children eligible, approached and consented to both trials. Unit 2 did not record data on approaches or refusals, but consented a total of 14 children to CHiP during the overlap period, one of whom was successfully co-enrolled to CATCH.

**Figure 3:**
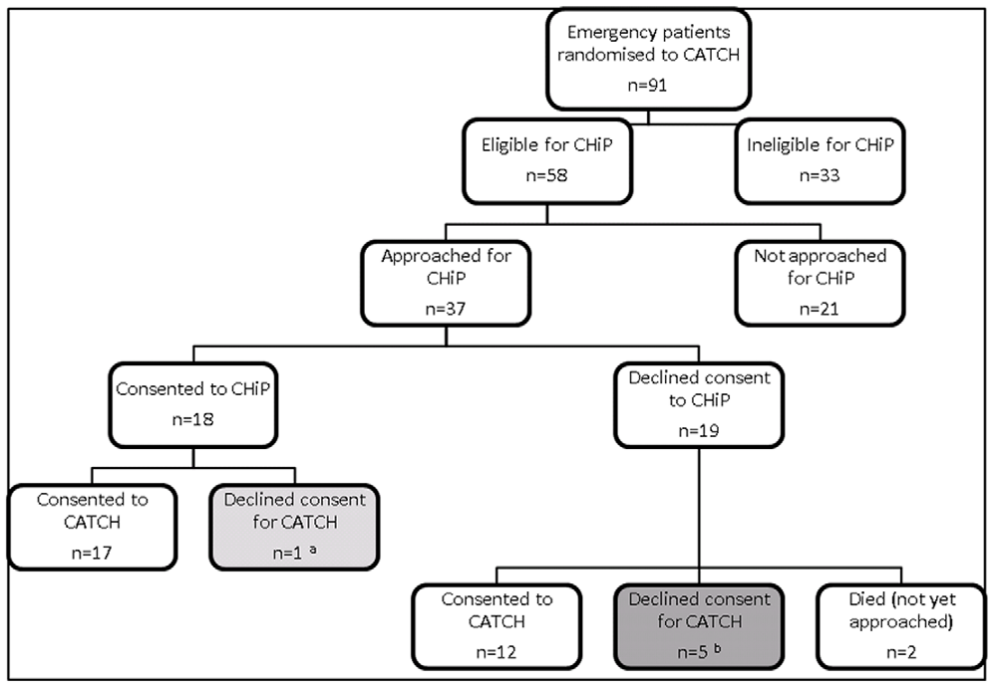
Emergency recruitment to CATCH and co-enrolment with CHiP.

Figure 3 shows the number of co-enrolments for emergency patients randomised to CATCH at Unit 1. In total, 35 sets of parents/guardians were approached for both studies (including 3 who declined consent to CHiP due to not wanting to take part in *any* research and so were not actually approached for CATCH). Two patients who had declined consent to CHiP died before being approached for CATCH and parents are being followed-up for deferred consent as per protocol. The consent rate was 82% (29/35) for CATCH and 51% (18/35) for CHiP.

Recruitment rates for parents approached at the same PICU for a single study were 78% (47/60, CATCH emergency admissions) and 51% (192/377, CHiP), suggesting that co-enrolment did not affect recruitment. This is supported by the reasons given for declining consent to CATCH after having been approached for CHiP (Table 1). These reasons were those given by parents to research nurses when asked, and may not provide in-depth understanding of parental decisions.

**Table 1:** Reasons for refusal to consent.

Event	Reason for refusal
^a^Consented to CHiP, declinedconsent to CATCH	Consented to CHIP but approached for CATCH after Social services becameinvolved for suspected intentional injury (n = 1)
	Didn’t want to take part in any research (n = 3)
^b^Declined consent to CHiP,declined consent to CATCH	Felt that the child had been through too much already (n = 1)
	No reason given (n = 1)

Parents of children admitted to this PICU whilst both trials were on-going were more likely to consent to CATCH than CHiP. Possible reasons for this difference are that emergency admissions approached for CATCH had already been randomised to a trial CVC - so parents were consenting to data collection only – and that CATCH posed minimal risk to children compared with greater risks involved with CHiP. For CHiP, consent could mean a change in treatment plan. Secondly, consent for CHiP was sought prior to randomisation, and the limited time to consider study participation might have deterred some parents. This limited time frame also meant that some eligible children were not approached for CHiP.

Of 11 elective patients consented to CATCH, two later became eligible for CHiP (after surgery). Neither of these children were approached for CHiP in time and so no elective patients were co-enrolled.

### Consent Process

The research teams for both trials in Unit 1 worked together to facilitate co-enrolment. The same team approached the parents about both trials if they already had a rapport with the parents, so that families were not meeting too many new faces. Communication between teams was important to minimise approaches to parents – if a parent declined to consent for CHiP due to not wanting to take part in *any* kind of research, they were not approached for CATCH.

Parents were approached for CATCH after they had made a decision about whether or not to enrol for CHiP in all but two situations: 1) when children became eligible for CHiP after having been approached for CATCH, and 2) on two occasions, research nurses felt it was appropriate to approach for both trials together (simultaneous consent). Research nurses found it easiest to consent separately for each trial although simultaneous consent was successful on both occasions when this was attempted. Feedback from research nurses indicated that some parents found the responsibility of having to make a decision about consent to a trial too burdensome at their time of stress, and would have preferred that the decision was made by the medical team. This was reflected in the high recruitment rate for emergency CATCH patients, who would already have had the intervention when consent was sought.

In Unit 2, research nurses expressed that they found it difficult to talk to parents about both trials at the same time, and the parents of the child that did co-enrol were approached on different days. However, research nurses at this unit felt that co-enrolment is important and co-enrolment with CATCH and other on-going trials is continuing.

## Discussion

Most PICUs refused to co-enrol citing concerns about jeopardising recruitment to CHiP (which was already running), and too much information for parents. These concerns were not supported by evidence from the PICUs that did co-enrol. Consent rates for children approached for co-enrolment were similar to overall consent rates, and reasons for refusal did not mention information overload. However, parents had a clear preference for the study that did not involve a change in treatment, concerned interventions already in routine use, and posed minimal risk to patients. Although based mainly on findings from one PICU, our experience with parental decisions in CATCH and CHiP is supported by previous small studies showing that the majority of parents are willing to be approached for multiple trials [19], [20].

The perceived ethics restrictions and concerns about recruitment targets were barriers to PICU decisions to enrol children into both trials. In the one unit that did co-enrol, the process for consenting to two trials was determined by two main issues. First, priority was given to CHiP recruitment so that sample size targets could be met, which meant that deferred consent for CATCH was delayed further whilst parents were given time to consider participating in CHiP. Second, ethics restrictions on modification of existing patient information leaflets, which meant that research nurses were unable to present simple information for both trials together thereby minimising the burden on parents.

Research evidence shows that presenting comprehensive but concise information may increase the level of understanding and the likelihood of true informed consent [21]. Strategies for the whole consent process (information sheets and how to approach patients) when more than one trial is available need to be carefully developed, piloted and modified if necessary. Ethics approval of combined forms of patient information also needs to be flexible and rapid, to avoid impeding study start up or recruitment.

The decision to allow co-enrolment needs careful consideration of the trials in question and potential impact on results, possible interaction between therapies, internal and external validity and safety (e.g. maximum blood volume allowed for research) [22], [23], [24]. For large pragmatic trials such as CATCH, overlap with other trials is one aspect of the heterogeneity of practice within PICUs, whereas co-enrolment in smaller efficacy studies may not be appropriate. As CATCH is a low-risk trial, we are not able to make assumptions about co-enrolment for studies where parents are asked to take more than one moderate risk.

Clinicians’ and researchers’ concerns about co-enrolment centre mainly on the potential burden of multiple trials on parents, yet others argue that parents have the right to be made aware of all possible options for their child [25]. Without co-enrolment, the priority given to trials is determined by chief investigators or PICUs rather than patient choice. The little research that has been conducted on parental opinions suggests that parents are supportive of co-enrolment (in neonates), and that the possibility of any beneficial new treatment and the general benefits of being involved in a trial are attractive to parents [19], [20]. The number of trials offered to parents should not be overwhelming (one survey found that parents of neonates on average thought that two trials at a time would be acceptable) [20]. However, defining a maximum number of trials has issues - some patients may not be offered entry into a study if they had reached their maximum, potentially jeopardising recruitment targets or introducing bias into analysis [26].

Our examination of PICU and parental decisions in recruiting to two large RCTs has highlighted the importance, acceptability and success of recruitment into multiple studies, but also that barriers to co-enrolment remain. Our experience with CATCH and CHiP should encourage both ethics committees and clinicians to feel confident about the acceptability and feasibility of co-enrolment and to develop strategies that minimise burden on parents but allow the capacity for important research in PICU to continue to increase [11], [12].
